# Prognostic value of preoperative serum alkaline phosphatase for predicting 3-year mortality in patients undergoing kidney transplantation: A retrospective study

**DOI:** 10.1371/journal.pone.0273662

**Published:** 2022-08-29

**Authors:** Hei Jin Yoon, Da Eun Ko, Sang Beom Nam, Young Song, Byung Hwan Yun, Sung Yeon Ham

**Affiliations:** 1 Department of Anesthesiology and Pain Medicine, Yonsei University College of Medicine, Seoul, Republic of Korea; 2 Anesthesia and Pain Research Institute, Yonsei University College of Medicine, Seoul, Republic of Korea; Nazarbayev University School of Medicine, KAZAKHSTAN

## Abstract

Serum alkaline phosphatase (ALP) levels are related to high-turnover bone disease and reflect vascular calcification and inflammation. ALP has been reported to have a prognostic impact in various cohorts including chronic kidney disease. This study investigated whether preoperative serum ALP level could be used for predicting mortality in patients undergoing kidney transplantation. We retrospectively reviewed 1,718 patients who underwent kidney transplantation between November 2005 and June 2017. Finally, 1,533 patients who met the inclusion criteria were classified into tertiles based on preoperative serum ALP level (< 51, 51–72, > 72 IU/L). The incidence of mortality was compared among the three tertiles, and a stepwise logistic regression analysis was performed to evaluate the predictors for mortality. The incidence of 3-year mortality was the highest in the third tertile (1.0% vs. 2.5% vs. 4.4% in the first, second, and third tertile, respectively, p = 0.003). The third tertile of ALP level (odds ratio [OR] 1.855, 95% CI 1.192–2.886, p = 0.006), age (OR 1.052, 95% CI 1.022–1.082, p = 0.011), and history of hypertension (OR 0.401, 95% CI 0.210–0.765, p = 0.006) remained as independent predictors of mortality. Preoperative serum ALP level was significantly higher in the non-survivor group than in the survivor group (58.00 [44.00–76.00] vs. 75.00 [56.25–113.00], p = 0.003). The optimal cut-off value of serum ALP to predict 3-year mortality was 71 IU/L (area under the curve 0.636, 95% CI 0.554–0.719, p = 0.003). Therefore, preoperative serum ALP level was an independent predictor of 3-year mortality in patients undergoing kidney transplantation.

## Introduction

The definite treatment for end-stage renal disease is kidney transplantation. Despite the development and recent advances in kidney transplantation, patients with chronic kidney disease (CKD) are highly prone to have a poor prognosis due to complications from CKD itself and various comorbidities. However, there are a few reports on risk stratifications for postoperative morbidity and mortality in patients undergoing kidney transplantation.

Serum alkaline phosphatase (ALP) is frequently used as a biomarker of high-turnover bone disease, and is known to be related to vascular calcification and inflammation [1, 2]. In this context, ALP has been demonstrated to have a prognostic impact among older adults [3], and patients with coronary artery disease [4–6], and stroke [7]. Furthermore, ALP has been used to monitor bone metabolism and has a prognostic impact in patients with renal insufficiency whose mineral metabolism is altered [8]. Several studies have reported the association between ALP and mortality in patients with CKD and those on dialysis [9–12]. ALP predicts the prognosis of patients with CKD with altered mineralization and mineral metabolism changes after kidney transplantation. However, whether ALP may function as a predictor of prognosis in patients with CKD undergoing kidney transplantation has never been studied before.

Therefore, we aimed to conduct a retrospective study to investigate the association between preoperative serum ALP level and mortality in patients undergoing kidney transplantation.

## Materials & methods

### Study population

**Fig 1:**
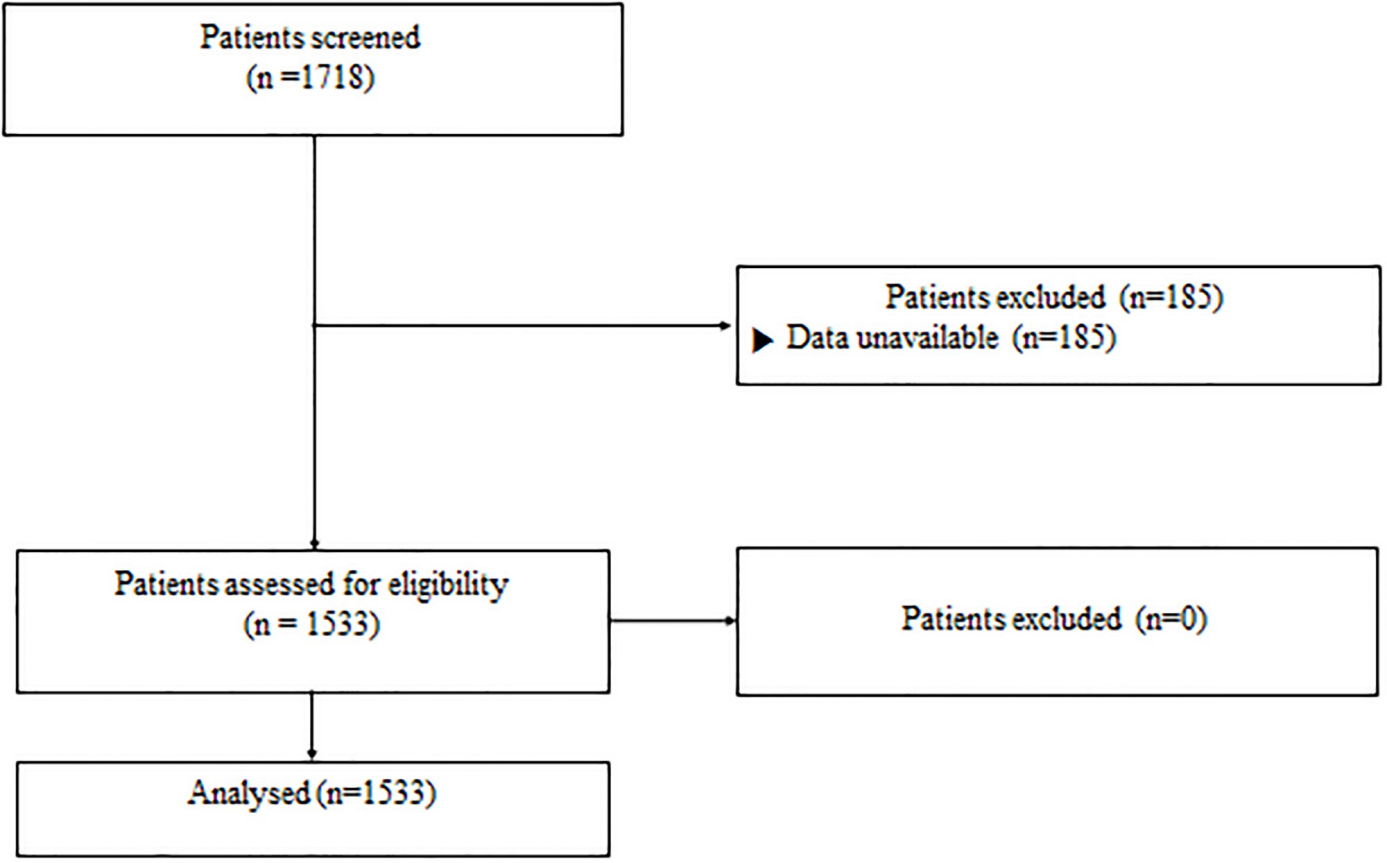
Flowchart of study enrolment.

This study was approved by our Institutional Review Board (IRB protocol No. 3-2021-0129), and the need for informed consent was waived due to the retrospective nature of the study. All data were fully anonymized before we accessed. We retrospectively reviewed the electronic medical records of patients who underwent kidney transplantation from November 2005 to June 2017 at the Severance Hospital. Out of 1,718 patients, 185 patients were excluded owing to data unavailability. A total of 1,533 patients were analyzed (Fig 1). Patients were divided into tertiles based on preoperative ALP levels (1^st^ tertile: ALP < 51 IU/L [n = 507]; 2^nd^ tertile ALP 51–72 IU/L [n = 522]; and 3^rd^ tertile ALP > 72 IU/L [n = 504]).

### Study endpoints

The primary outcome of this study included the incidence of 3-year mortality after kidney transplantation. Secondary outcomes included the incidence of graft failure, new-onset diabetes after kidney transplantation (NODAT), myocardial infarction (MI), stroke, and acute kidney injury (AKI). Stroke was defined as the presence of newly developed neurological deterioration due to embolic, thrombotic, or hemorrhagic brain injury. AKI was defined as an increase in serum creatinine ≥ 0.3 mg/dL, an increase to 150% of baseline value, or urine output <0.5 ml/kg/h for ≥ 6 h consecutively within 48 h after kidney transplantation.

### Other assessments

Demographics included sex, age, height, weight, history of smoking, hypertension, diabetes mellitus, coronary artery occlusive disease, congestive heart failure, peripheral artery occlusive disease, chronic obstructive pulmonary disease, and liver disease. Laboratory data including the levels of ALP, albumin, hemoglobin, creatinine, alanine aminotransferase (ALT), aspartate aminotransferase (AST), total bilirubin, parathyroid hormone (PTH), calcium, phosphorus, and C-reactive protein were recorded.

Postoperatively, clinical outcomes including graft failure, NODAT, MI, stroke, postoperative renal replacement therapy (RRT) requirement, AKI, pulmonary complication, and mortality were assessed.

### Statistical analyses

All statistical analyses were performed using the Statistical Package for Social Sciences version 23 (IBM Corp, Armonk, NY, USA). After normality assessment using the Kolmogorov-Smirnov test, the independent t-test or Mann-Whitney test was used to compare continuous variables. To compare the three groups, one-way ANOVA or Kruskal–Wallis test was used, as appropriate. Categorical variables were compared using the chi-squared or Fisher’s exact test. Results are presented as means ± standard deviation (SD) for normally distributed data, medians (IQR) for skewed data, or number of patients (%). We performed logistic regression analysis to evaluate predictors of 3-year mortality after kidney transplantation with known risk factors and variables with p<0.2 depending on patient mortality. For multivariate analysis, a stepwise selection method was used and variables with p<0.2 in univariate analysis were selected. Predictability was expressed as odds ratio (OR) and 95% CI. Receiver-operating characteristic (ROC) curve analysis was used to determine the optimal cut-off value of preoperative serum ALP showing the best discriminatory capacity to predict postoperative 3-year mortality. The optimal cut-off value was defined as the serum ALP level showing the greatest sum of sensitivity and specificity. Multivariate logistic regression analysis was performed to find variables associated with ALP higher than the cut-off value we found (≥71 IU/L). A p-value < 0.05 was considered statistically significant.

## Results

A total of 1,718 patients were screened, and 1,533 patients were finally included for the analysis (Fig 1). Patients were classified into tertiles based on the preoperative ALP level (1^st^ tertile: ALP < 51 IU/L [n = 507], 2^nd^ tertile ALP 51–72 IU/L [n = 522], and 3^rd^ tertile ALP > 72 IU/L [n = 504]). Table 1 shows the baseline characteristics of patients across tertiles. The number of female patients (45.8% vs. 36.6% vs. 43.1%, p = 0.009), height (164.69±8.52 cm vs. 165.51±8.21 cm vs. 162.40±11.97 cm, p<0.001), and weight (61.30±11.52 kg vs. 63.86±12.34 kg vs. 59.56±13.86 kg, p<0.001) were significantly different among the three groups. The proportion of patients with a history of hypertension (74.0% vs. 70.7% vs. 56.7%, p*<*0.001), diabetes mellitus (21.5% vs. 26.8% vs. 17.5%, p = 0.001), chronic obstructive pulmonary disease (0.2% vs. 0% vs. 0.8%, p = 0.031), and liver disease (0.2% vs. 1.3% vs. 1.8%, p = 0.045) was significantly different among the three groups. Preoperative values of albumin (3.83±0.50 vs. 3.88±0.54 vs. 3.96±0.58 g/dL, p<0.001), ALT (13.35±8.30 vs. 15.48±11.36 vs. 17.68±14.46 IU/L, p<0.001), AST (15.49±7.92 vs. 16.68±9.07 vs. 19.14±11.17 IU/L, p<0.001), calcium (8.73±0.87 vs. 8.88±0.96 vs. 8.83±0.99 mg/dL, p = 0.034), PTH (235.97±184.56 vs. 283.72±208.37 vs. 413.28±374.97 pg/mL, p<0.001), and hemoglobin (10.15±1.73 vs. 10.55±1.62 vs. 10.67±1.74 g/dL, p<0.001) were also significantly different among the ALP tertiles.

**Table 1:** Baseline characteristics of patients who underwent kidney transplantation.

	First tertile	Second tertile	Third tertile	P-value
	(n = 507)	(n = 522)	(n = 504)	
Female sex	232 (45.8%)	191 (36.6%)	217 (43.1%)	0.009*
Age (years)	43.51±11.24	44.84±11.88	43.42±14.34	0.112
Height (cm)	164.69±8.52	165.51±8.21	162.40±11.97	<0.001*
Weight (kg)	61.30±11.52	63.86±12.34	59.56±13.86	<0.001*
Smoking	64 (12.6%)	69 (13.2%)	61 (12.1%)	0.865
HTN	375 (74.0%)	369 (70.7%)	286 (56.7%)	<0.001*
DM	109 (21.5%)	140 (26.8%)	88 (17.5%)	0.001*
CAOD	25 (4.9%)	31 (5.9%)	26 (5.2%)	0.752
CHF	0	2 (0.4%)	1 (0.2%)	0.662
PAOD	1 (0.2%)	2 (0.4%)	0	0.777
COPD	1 (0.2%)	0	4 (0.8%)	0.031*
Liver disease	1 (0.2%)	7 (1.3%)	9 (1.8%)	0.045*
Preoperative ALP (IU/L)	38.86±8.34	60.25±6.41	116.88±88.02	<0.001*
Preoperative albumin (g/dL)	3.83±0.50	3.88±0.54	3.96±0.58	<0.001*
Preoperative Hb (g/dL)	10.15±1.73	10.55±1.62	10.67±1.74	<0.001*
Preoperative Cr (mg/dL)	7.74±3.35	8.10±3.60	7.97±3.46	0.247
Preoperative ALT (IU/L)	13.35±8.30	15.48±11.36	17.68±14.46	<0.001*
Preoperative AST (IU/L)	15.49±7.92	16.68±9.07	19.14±11.17	<0.001*
Preoperative T.bil (mg/dL)	0.34±0.14	0.34±0.16	0.40±1.01	0.462
Preoperative PTH (pg/mL)	235.97±184.56	283.72±208.37	413.28±374.97	<0.001*
Preoperative Ca (mg/dL)	8.73±0.87	8.88±0.96	8.83±0.99	0.034*
Preoperative P (mg/dL)	5.15±1.62	5.16±1.61	5.16±1.54	0.996
Preoperative CRP (mg/L)	4.21±9.78	3.78±8.77	5.41±12.38	0.237

Values are presented as the mean±standard deviation, or number of patients (%)

HTN: hypertension, DM: diabetes mellitus, CAOD: coronary artery occlusive disease, CHF: congestive heart failure, PAOD: peripheral arterial occlusive disease, COPD: chronic obstructive pulmonary disease, ALP: alkaline phosphatase, Hb: hemoglobin, Cr: creatinine, ALT: alanine transaminase, AST: aspartate transaminase, T.bil: total bilirubin, PTH: parathyroid hormone, Ca: calcium, P: phosphorous, CRP: C-reactive protein.

The incidence of postoperative complications including the requirement of RRT (0.4% vs. 0.8% vs. 2.4%, p = 0.008) and AKI (0.6% vs. 1.1% vs. 2.4%, p = 0.043) was significantly higher in the third tertile than in the lower two tertiles. The incidence of 1-year (0.6% vs. 1.3% vs. 3.0%, p = 0.009) and 3-year mortality (1.0% vs. 2.5% vs. 4.4%, p = 0.003) was the highest in the third tertile group while the incidence of short-term mortality was similar among the groups (Table 2).

**Table 2:** Postoperative morbidity and mortality of patients.

	First tertile	Second tertile	Third tertile	P-value
	(n = 507)	(n = 522)	(n = 504)	
ICU day (days)	0.29±3.63	0.26±1.38	0.57±3.94	0.243
HOD (days)	25.40±14.48	25.71±11.01	26.52±14.14	0.389
Graft failure	3 (0.6%)	2 (0.4%)	4 (0.8%)	0.591
NODAT	11 (2.2%)	11 (2.1%)	8 (1.6%)	0.763
MI	4 (0.8%)	0	1 (0.2%)	0.051
Stroke	1 (0.2%)	1 (0.2%)	2 (0.1%)	1.000
Postop RRT	2 (0.4%)	4 (0.8%)	12 (2.4%)	0.008*
Postop AKI	3 (0.6%)	6 (1.1%)	12 (2.4%)	0.043*
In-hospital mortality	1 (0.2%)	3 (0.6%)	7 (1.4%)	0.073
1-month mortality	2 (0.4%)	3 (0.6%)	4 (0.8%)	0.717
6-month mortality	2 (0.4%)	6 (1.1%)	10 (2.0%)	0.064
1-year mortality	3 (0.6%)	7 (1.3%)	15 (3.0%)	0.009*
3-year mortality	5 (1.0%)	13 (2.5%)	22 (4.4%)	0.003*

Values are presented as the mean±standard deviation, or number of patients (%)

ICU: intensive care unit, HOD: hospital stay, NODAT: new-onset diabetes after kidney transplantation, MI: myocardial infarction, RRT: renal replacement therapy, AKI: acute kidney injury.

Using logistic regression analysis, the ALP level of the third tertile, age, and hypertension showed a difference of p<0.2 for predicting 3-year mortality of patients. The third tertile of ALP level (OR = 1.855, 95% CI 1.192–2.886, p = 0.006), age (OR = 1.052, 95% CI 1.022–1.082, p = 0.011), and history of hypertension (OR = 0.401, 95% CI 0.210–0.765, p = 0.006) remained as independent predictors of 3-year mortality in multivariate analysis (Table 3).

**Table 3:** Logistic regression analysis for predictors of 3-year mortality of patients after kidney transplantation.

	Univariate OR (CI)	P-value	Multivariate OR (CI)	P-value
Age	1.052 (1.021–1.083)	0.001	1.052 (1.022–1.082)	0.001
Smoking	1.481 (0.646–3.397)	0.353		
HTN	0.389 (0.207–0.732)	0.003	0.401 (0.210–0.765)	0.006
DM	0.884 (0.404–1.938)	0.759		
CAOD	0.930 (0.220–3.922)	0.921		
PTH	1.000 (0.999–1.002)	0.652		
ALP tertile	2.045 (1.327–3.152)	0.001	1.855 (1.192–2.886)	0.006

Values are presented as odds ratio (95% confidential interval).

HTN: hypertension, DM: diabetes mellitus, CAOD: coronary artery occlusive disease, ALP: alkaline phosphatase.

Table 4 shows the baseline characteristics and outcome variables of patients stratified according to 3-year mortality. Non-survivors were significantly older than survivors (47.00 [36.00–55.00] vs. 52.50 [47.25–62.00] years, p<0.001) and the proportion of patients with hypertension was significantly higher among the survivors than among the non-survivors (67.8% vs. 45.0%, p = 0.002). Preoperative ALP was significantly different between the two groups (58.00 [44.00, 76.00] vs. 75.00 [56.25, 113.00] IU/L, p = 0.003). Preoperative values of ALT (12.00 [9.00–18.00] vs. 18.50 [12.00–38.25] IU/L, p = 0.010) and AST (16.00 [12.00–20.00] vs. 18.50 [15.25–33.75] IU/L, p<0.001) were significantly different between survivors and non-survivors. The duration of ICU (0.00 [0.00–0.00] days vs. 0.00 [0.00–1.00] days, p*<*0.001) and hospital stay (22.00 [20.00–26.00] days vs. 24.00 days [20.50–54.25], p = 0.034) was significantly longer in the non-survivors compared with the survivors. The incidence of graft failure (0.5% vs. 5.0%, p = 0.021), MI (0.2% vs. 5.0%, p = 0.006), requirement of postoperative RRT (0.9% vs. 10.0%, p = 0.001), and AKI (1.1% vs. 10.0%, p = 0.002) was significantly higher in non-survivors than in survivors.

**Table 4:** Baseline characteristics and outcome variables of patients stratified based on 3-year mortality.

	Survivor (n = 1,493)	Non-survivor (n = 40)	P-value
Female sex	627 (42.0%)	13 (32.5%)	0.229
Age (years)	47.00 (36.00, 55.00)	52.50 (47.25, 62.00)	<0.001*
Height (cm)	164.50 (157.98, 170.93)	163.50 (160.95, 167.38)	0.186
Weight (kg)	60.60 (52.46, 69.03)	65.85 (55.55, 73.80)	0.725
Smoking	187 (12.5%)	7 (17.5%)	0.350
HTN	1,012 (67.8%)	18 (45.0%)	0.002*
DM	329 (22.0%)	8 (20.0%)	0.759
CAOD	80 (97.6%)	2 (5.0%)	1.000
CHF	3 (0.2%)	0	1.000
PAOD	3 (0.2%)	0	1.000
COPD	5 (0.3%)	0	1.000
Liver disease	16 (1.1%)	1 (2.5%)	0.364
Preoperative ALP (IU/L)	58.00 (44.00, 76.00)	75.00 (56.25, 113.00)	0.003*
Preoperative albumin (g/dL)	3.80 (3.50, 4.10)	3.80 (3.25, 4.05)	0.427
Preoperative Hb (g/dL)	10.40 (9.73, 11.50)	10.95 (9.90, 12.05)	0.931
Preoperative Cr (mg/dL)	6.53 (5.13, 9.02)	7.73 (5.73, 10.97)	0.229
Preoperative ALT (IU/L)	12.00 (9.00, 18.00)	18.50 (12.00, 38.25)	0.010*
Preoperative AST (IU/L)	16.00 (12.00, 20.00)	18.50 (15.25, 33.75)	<0.001*
Preoperative T.bil (mg/dL)	0.30 (0.20, 0.40)	0.40 (0.30, 0.40)	0.067
Preoperative PTH (pg/mL)	229.10 (117.70, 364.00)	200.60 (123.45, 367.15)	0.736
Preoperative Ca (mg/dL)	8.70 (8.20, 9.30)	8.85 (8.45, 9.20)	0.512
Preoperative P (mg/dL)	4.80 (3.90, 5.80)	4.45 (3.65, 7.00)	0.191
Preoperative CRP (mg/L)	1.11 (0.60, 3.31)	1.65 (0.63, 4.58)	0.318
ICU stay (days)	0.00 (0.00, 0.00)	0.00 (0.00, 1.00)	<0.001*
HOD (days)	22.00 (20.00, 26.00)	24.00 (20.50, 54.25)	0.034*
Graft failure	7 (0.5%)	2 (5.0%)	0.021*
NODAT	29 (1.9%)	1 (2.5%)	0.551
MI	3 (0.2%)	2 (5.0%)	0.006*
Stroke	2 (0.1%)	0	1.000
Postop RRT	14 (0.9%)	4 (10.0%)	0.001*
Postop AKI	17 (1.1%)	4 (10.0%)	0.002*

Values are presented as the mean±standard deviation, median (interquartile range), or number of patients (%)

HTN: hypertension, DM: diabetes mellitus, CAOD: coronary artery occlusive disease, CHF: congestive heart failure, PAOD: peripheral arterial occlusive disease, COPD: chronic obstructive pulmonary disease, ALP: alkaline phosphatase, Hb: hemoglobin, Cr: creatinine, ALT: alanine transaminase, AST: aspartate transaminase, T.bil: total bilirubin, PTH: parathyroid hormone, Ca: calcium, P: phosphorous, CRP: C-reactive protein, ICU: intensive care unit, HOD: hospital stay, NODAT: new-onset diabetes after kidney transplantation, MI: myocardial infarction, RRT: renal replacement therapy, AKI: acute kidney injury.

**Fig 2:**
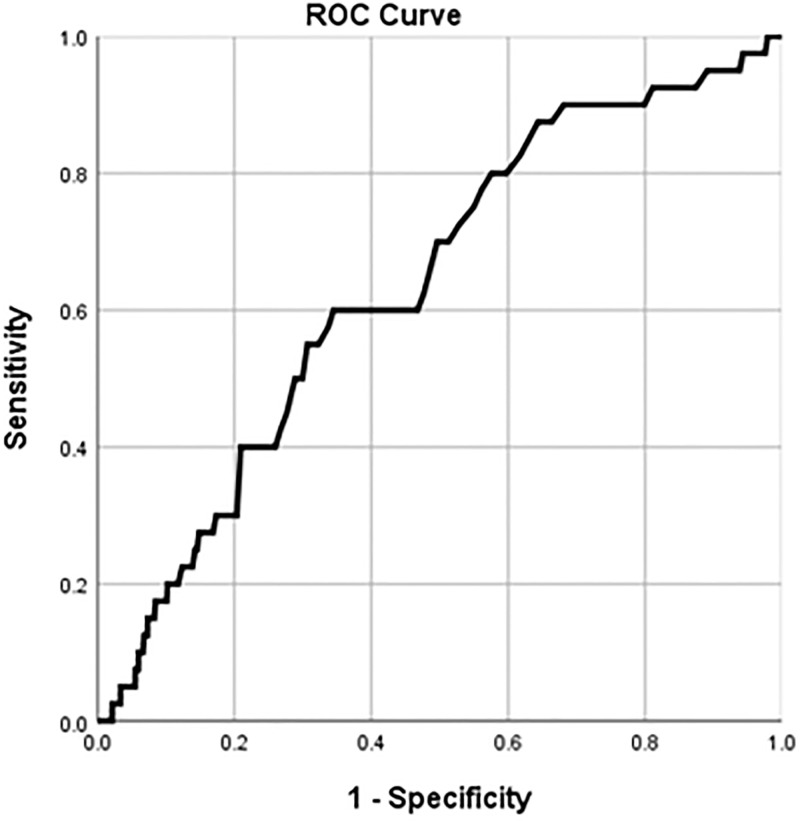
Combined receiver-operating characteristic curve of preoperative serum ALP levels for the incidence of 3-year mortality. The area under the curve = 0.636 and *p*-value = 0.003 are observed below the line showing the serum ALP level with a 95% confidence interval of 0.554–0.719. The optimal cut-off value of serum ALP that predicted the incidence of 3-year mortality was 71 IU/L, with a sensitivity and specificity of 60.0% and 65.5%, respectively.

ROC curve of preoperative serum ALP levels for predicting 3-year mortality after kidney transplantation demonstrated an area under the curve of 0.636. (95% CI 0.554–0.719, p = 0.003). The optimal cut-off value of preoperative ALP that predicted 3-year mortality was 71 IU/L with a sensitivity and specificity of 60.0% and 65.5%, respectively (Fig 2).

Table 5 shows postoperative morbidity and mortality stratified according to the cut-off value. The incidence of postoperative RRT requirement (0.6% vs. 2.2%, p = 0.005), AKI (0.9% vs. 2.2%, p = 0.034), 1-year (1.0% vs. 2.8%, p = 0.009), and 3-year mortality (1.6% vs. 4.5%, p = 0.001) was significantly higher in the higher ALP group than in the lower ALP group.

**Table 5:** Postoperative morbidity and mortality of patients stratified based on cut-off values of ALP.

	ALP < 71 (n = 994)	ALP ≥71 (n = 539)	P-value
ICU day	0.25±2.70	0.59±3.90	0.076
HOD	25.51±12.93	26.54±13.89	0.157
Graft failure	5 (0.5%)	4 (0.7%)	0.728
NODAT	20 (2.0%)	10 (1.9%)	0.832
MI	4 (0.4%)	1 (0.2%)	0.662
Stroke	2 (0.2%)	0	0.544
Postop RRT	6 (0.6%)	12 (2.2%)	0.005*
Postop AKI	9 (0.9%)	12 (2.2%)	0.034*
In-hospital mortality	4 (0.4%)	7 (1.3%)	0.059
1-month mortality	5 (0.5%)	4 (0.7%)	0.728
6-month mortality	8 (0.8%)	10 (1.9%)	0.068
1-year mortality	10 (1.0%)	15 (2.8%)	0.009*
3-year mortality	16 (1.6%)	24 (4.5%)	0.001*

Values are presented as the mean±standard deviation, or number of patients (%)

ICU: intensive care unit, HOD: hospital stay, NODAT: new-onset diabetes after kidney transplantation, MI: myocardial infarction, RRT: renal replacement therapy, AKI: acute kidney injury.

In a multivariate logistic regression analysis to find possible associated factor with higher ALP, higher PTH was a predictor of higher ALP (≥71 IU/L) (Table 6).

**Table 6:** Logistic regression analysis for predictors of higher ALP (≥71 IU/L).

	Univariate OR (CI)	P-value	Multivariate OR (CI)	P-value
Age	0.996 (0.998–1.004)	0.347		
ALT	1.027 (1.017–1.037)	<0.001	1.018 (0.998–1.039)	0.083
AST	1.036 (1.024–1.048)	<0.001	1.017 (0.992–1.042)	0.183
T.bil	1.212 (0.901–1.630)	0.204		
PTH	1.002 (1.001–1.003)	<0.001	1.002 (1.001–1.003)	<0.001
Ca	1.048 (0.938–1.172)	0.407		
P	0.989 (0.926–1.056)	0.739		
CRP	1.011 (0.997–1.025)	0.134		

Values are presented as odds ratio (95% confidential interval).

ALP: alkaline phosphatase, ALT: alanine transaminase, AST: aspartate transaminase, T.bil: total bilirubin, PTH: parathyroid hormone, Ca: calcium, P: phosphorus, CRP: C-reactive protein

## Discussion

In this retrospective study, we demonstrated that preoperative serum ALP level could predict 3-year mortality of patients after kidney transplantation. The incidence of 1-year and 3-year mortality after kidney transplantation was the highest in the third tertile of ALP, whereas the incidence of 1-month and 6-month mortality among tertiles showed no significant differences. Moreover, the third tertile of ALP was demonstrated to be an independent predictor for developing 3-year mortality after kidney transplantation.

ALP is a hydrolase enzyme that dephosphorylates various molecules expressed in the bone, liver, placenta, and kidneys. Serum ALP is a biochemical marker of bone turnover or liver disease. There are four ALP isozymes (tissue-nonspecific isozyme, intestinal-type, placental-type, and placental-like). Among these isozymes, tissue-nonspecific ALP is the most abundant isoform and is involved in skeletal mineralization [13]. Since mineral metabolism is commonly altered in patients with CKD, ALP is frequently used to monitor bone metabolism associated with renal insufficiency [8].

Disorders related to calcium, phosphorous, and parathyroid hormone (PTH) are commonly observed in patients with CKD. Along with renal osteodystrophy, secondary hyperparathyroidism is common [14], and it may be associated with abnormal mineral metabolism leading to vascular calcification and poor prognosis [15–17]. Serum ALP levels have been demonstrated to be associated with mortality in patients with renal failure [11, 12, 18]. Serum ALP ≥120 IU/L was reported to be associated with mortality among patients undergoing hemodialysis [18]. The highest ALP quartile was associated with higher risk of mortality in patients undergoing peritoneal dialysis [12]. Although the association of abnormal mineral metabolism and CKD have been well established and the prognostic role of ALP in these patients has been demonstrated, the prognostic role of ALP in patients after kidney transplantation has not been investigated before. Further, bone turnover and abnormal bone mineralization showed changes even after kidney transplantation [19, 20]. Thus, the strength of our study is that we investigated the prognostic role of ALP in patients undergoing kidney transplantation, rather than patients with CKD.

The mechanisms responsible for the association between elevated ALP levels and mortality after kidney transplantation are unclear. The first possible explanation is that ALP is a marker of high-turnover bone disease. Elevated serum PTH induces bone resorption and this can be manifested by elevated bone ALP levels [21]. Similarly, the PTH level showed significant differences between the groups according to the ALP tertile and higher PTH was an independent predictor of higher ALP (≥71 IU/L) in the logistic regression analysis in the current study. A previous study demonstrated that mortality prediction by ALP was likely due to renal osteodystrophy [22]. Further, secondary hyperparathyroidism was associated with increased mortality in patients with CKD [23]. From these results, it can be inferred that ALP levels are increased due to renal osteodystrophy and secondary hyperparathyroidism in patients with CKD, and it can be associated with increased mortality. Furthermore, ALP levels have been demonstrated to be related with vascular calcification. A previous *in vitro* study revealed that vascular damage induces expression of tissue-nonspecific ALP and that ALP can promote calcification by hydrolyzing inorganic pyrophosphates [24]. An association between ALP and vascular calcification was also demonstrated in previous clinical studies [1, 25]. Another possibility is the association of ALP and systemic inflammation. Elevated ALP levels were shown to be related to elevated C-reactive protein levels, indicative of systemic inflammation [26]. Further, inflammatory stimuli could lead to cellular responses, which could increase ALP expression [13]. Along these lines, a previous study reported an association between higher serum ALP level and infection-related mortality in patients undergoing peritoneal dialysis [27].

The optimal cut-off value we found through ROC curve analysis was 71 IU/L, which was similar to the cut-off value of the highest ALP tertile (>72 IU/L), which was considerably lower than the value obtained in previous studies investigating the association between ALP and mortality in patients undergoing dialysis. ALP ≥120 IU/L was reported to be associated with mortality in patients undergoing hemodialysis [18]. Another study conducted in patients undergoing peritoneal dialysis showed that the highest tertile of ALP (>155 IU/L) was associated with an infection-related mortality [27]. A previous study on the association between pre-transplant ALP levels and mortality reported that ALP >120 IU/L was associated with an increased risk of mortality [28]. One notable difference is that the incidence of graft failure was significantly different according to the ALP level in the previous study, but not in our study. As calcium-phosphate-PTH homeostasis was found to be a predictor of graft rejection in previous studies [29, 30], there may be some correlation between elevated ALP, graft rejection, and increased mortality, which were not addressed in this study. This is because the cut-off value obtained in our study was not high enough to predict graft failure although it could predict mortality. In addition, since there is literature evidence demonstrating an increase in the ALP value itself rather than based on the specific cut-off value as a risk factor for mortality [12], the lower cut-off value obtained in the current study seems plausible.

Notably, ALP could predict long-term (1-year and 3-year) mortality rather than short-term mortality (1-month and 6-month) in this study. Considering that ALP levels are indicative of high-turnover bone disease, the cause of this prediction of long-term mortality may be a reflection of the severity of pre-existing disorders of bone and mineral metabolism. Because there are post-transplant bone disorders, which indicates pathologic processes occurring after transplantation that are superimposed on pre-existing disorders of bone and mineral metabolism secondary to kidney failure and/or diabetes mellitus [20]. Further, there have been several reports investigating postoperative changes in bone histomorphometry [19, 31] supporting this hypothesis, which is beyond the scope of this study.

The strengths of our study are that we found the prognostic value of ALP in patients undergoing kidney transplantation rather than patients with CKD and this was not reported before. Further, we included a relatively long-term follow-up period and found that ALP levels might predict long-term mortality other than short-term mortality, and we identified a relatively lower cut-off value, which was within normal limits.

The current study has several limitations. First, possible confounding factors such as liver disease, inflammatory status, and comorbidities were not adjusted. However, since ALP levels are affected by various factors, it is worthwhile to divide patients based on the ALP tertile and compare the characteristics accordingly. Second, the inability to distinguish various isoforms of tissue non-specific ALP such as bone-specific ALP may be another limitation. However, clinical utility of measuring various isoforms of ALP remains unclear [32]. Further, many previous studies on ALP targeted total ALP rather than specific isoforms [3, 4, 7, 12], and a previous study revealed that bone-specific ALP had weaker association with mortality than total ALP [9]. Lastly, there is a possibility that the recruited data are insufficient, resulting in a selection bias due to the retrospective nature of the study.

## Conclusions

In conclusion, preoperative serum ALP level was an independent predictor of 3-year mortality after kidney transplantation. This retrospective study also revealed that ALP levels can predict long-term mortality rather than short-term mortality after kidney transplantation, which was not investigated before. Our results suggest that ALP level has a prognostic impact on mortality of patients after kidney transplantation, and that the possible mechanisms for this might be associated with high-turnover bone disease, vascular calcification, and inflammation.
